# mSLAb – An open-source masked stereolithography (mSLA) bioprinter

**DOI:** 10.1016/j.ohx.2024.e00543

**Published:** 2024-06-10

**Authors:** Benedikt K. Kaufmann, Matthias Rudolph, Markus Pechtl, Geronimo Wildenburg, Oliver Hayden, Hauke Clausen-Schaumann, Stefanie Sudhop

**Affiliations:** a Center for Applied Tissue Engineering and Regenerative Medicine, Munich University of Applied Sciences, 80335 Munich, Germany; b Center for NanoScience – CeNS, Ludwig Maximilian University of Munich, 80539 Munich, Germany; c Heinz-Nixdorf-Chair of Biomedical Electronics, School of Computation, Information and Technology & Munich Institute of Biomedical Engineering, Technical University of Munich, TranslaTUM, Einsteinstraße 25, 81675 Munich, Germany

**Keywords:** Bioprinting, Tissue Engineering, mSLA, Bioprinter, Hydrogel, Stereolithography

## Abstract

3D bioprinting is a tissue engineering approach using additive manufacturing to fabricate tissue equivalents for regenerative medicine or medical drug testing. For this purpose, biomaterials that provide the essential microenvironment to support the viability of cells integrated directly or seeded after printing are processed into three-dimensional (3D) structures. Compared to extrusion-based 3D printing, which is most commonly used in bioprinting, stereolithography (SLA) offers a higher printing resolution and faster processing speeds with a wide range of cell-friendly materials such as gelatin- or collagen-based hydrogels and SLA is, therefore, well suited to generate 3D tissue constructs.

While there have been numerous publications of conversions and upgrades for extrusion-based printers, this is not the case for state-of-the-art SLA technology in bioprinting. The high cost of proprietary printers severely limits teaching and research in SLA bioprinting. With mSLAb, we present a low-cost and open-source high-resolution 3D bioprinter based on masked SLA (mSLA). mSLAb is based on an entry-level (€350) desktop mSLA printer (Phrozen Sonic Mini 4 K), equipped with temperature control and humidification of the printing chamber to enable the processing of cell-friendly hydrogels. Additionally, the build platform was redesigned for easy sample handling and microscopic analysis of the printed constructs. All modifications were done with off-the-shelf hardware and in-house designed 3D printed components, printed with the same printer that was being modified.

We validated the system by printing macroscopic porous scaffolds as well as hollow channels from gelatin-based hydrogels as representative structures needed in tissue engineering.

## Graphical abstract

**Figure:**
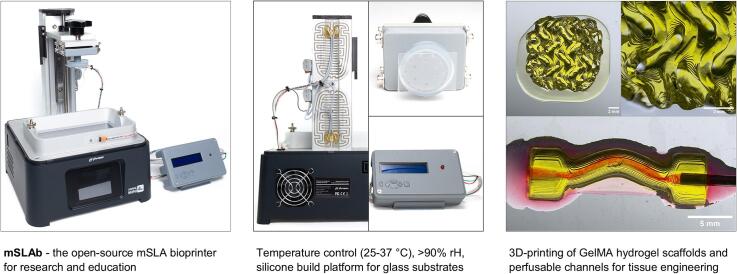

Specifications table

**Table:** 

Hardware name	mSLAb – an open-source mSLA bioprinter
Subject area	• Engineering and materials science • Medical • Biological sciences • Educational tools and open-source alternatives to existing infrastructure
Hardware type	Bioprinter
Closest commercial analog	CELLINK LUMEN X Gen 3
Open source license	CC BY-SA 4.0
Cost of hardware	€500
Source file repository	https://doi.org/10.17632/kxt5sks9zs.1

## 1 Hardware in context

Tissue engineering is an interdisciplinary field of research aiming to produce artificial tissue equivalents for regenerative medicine and medical drug testing by combining living cells with suitable materials in a 3D structure resembling natural tissue's architecture [1], [2]. Gelatin- or collagen-based hydrogels are frequently used for this purpose because, as proteins of the extracellular matrix (ECM), they represent the natural microenvironment of many cell types and support essential cellular processes like cell adhesion, migration, proliferation, and differentiation [3].

In the field of bioprinting, several additive manufacturing techniques are employed, differing significantly in their capabilities: Extrusion-based, jetting-based, and vat photopolymerization.

The most frequently used technology for generating artificial tissue constructs from ECM-based hydrogels is extrusion-based bioprinting (EBP). EBP creates three-dimensional structures by precisely layering the printing material through a nozzle or syringe in a predetermined pattern to construct tissue-like structures. Due to their simple construction, home-build EBP bioprinters can be realized easily, and numerous publications on conversions and upgrades for extrusion-based printers are available [4], [5], [6], [7]. EBP is well suited to generate larger 3D constructs from highly viscous materials. However, EBP also has disadvantages: The printability of proteinaceous, ECM-based hydrogels is limited: As soft hydrogels with poor mechanical stability, 3D geometries printed from ECM proteins show poor shape fidelity and easily collapse under their own weight. Compared to other techniques, the resolution is relatively low because it is limited by the nozzle diameter, which ranges typically from about 100 µm to a few mm [8], [9].

Much finer resolutions can be achieved by jetting-based bioprinting, which includes techniques like inkjet printing, microvalve-based printing, and laser-induced-forward-transfer (LIFT). In jetting-based printing, small droplets of printing material are propelled onto the substrate. The size of each utilized droplet can be controlled precisely and is not limited by any nozzle [10], [11]. However, it is contingent upon the availability of suitable low-viscosity bioinks at low concentrations (typically < 5 % (w/v)). The shear stress in the droplet formation process and during impact on the substrate is a primary factor limiting cell viability in printing cell-laden materials [10]. The LIFT process has a high cell viability and positioning accuracy [12], [13]. However, currently, LIFT is limited to predominately few-layer constructs and slow processing speeds (i.e., printed voxels over time) [14].

Vat photopolymerization uses a vat of liquid photopolymer, out of which the printed part is constructed layer by layer. A light source, usually a laser or a digital light projector, selectively cures and solidifies the photopolymer according to the digital design provided to the machine. It is known for its high spatial resolution (approx.10–50 µm for single-photon excitation and 0.2–1 µm for two-photon excitation) and finish quality, making it suitable for applications requiring fine features and smooth surfaces [15]. Common technologies of vat photopolymerization include Stereolithography (SLA), Digital Light Processing (DLP), masked SLA (mSLA), and two-photon polymerization (2PP). Stereolithography (SLA) uses a laser to cure photosensitive resin layer-by-layer, offering high precision and surface quality but suffering from slow speeds as the laser has to scan the geometry voxel by voxel. Digital Light Processing (DLP) accelerates the process by projecting entire layers at once, using a digital micromirror device, resulting in faster printing with potential resolution limitations due to pixelation. This is also true for the cost-efficient mSLA technology, where an LCD panel combined with high-power LED arrays is employed for illumination. This also enables multiplexing, i.e., the simultaneous printing of multiple structures without increasing the print time. This provides greater flexibility and efficiency and the ability to print numerous samples simultaneously, allowing better statistics for research. 2PP achieves sub-micron resolution by focusing a laser beam to polymerize resin solely at the focal point via two-photon excitation, which is ideal for specialized applications requiring extreme detail. Due to its slow processing speed and high complexity, 2PP is a niche technology restricted to specialized applications [16], [17], [18], [19].

All vat photopolymerization techniques can be used with functionalized protein-based biomaterials: In the presence of a photoinitiator and light, modified (ECM-based) proteins, such as gelatin methacryloyl (GelMA), can be crosslinked and structured into 3D shapes [20], [21], [22]. By varying the illumination time, the material's mechanical properties can be controlled [23]. However, the availability of suitable functionalized biomaterials and biocompatible photoinitiators currently limits light-based technologies [24].

Unfortunately, only a few proprietary high-priced SLA printers suited for bioprinting are available [25]. The lack of options and the high prices severely limit the use of this technology in academic teaching and research. Recent publications show complex and highly specialized bioprinting systems built from scratch by professional engineers, with advanced features such as automatically exchangeable material reservoirs [26], [27], [28], [29], [30]. However, there is no viable option for easy-to-use and cost-effective printers for research and teaching purposes.

**Fig. 1:**
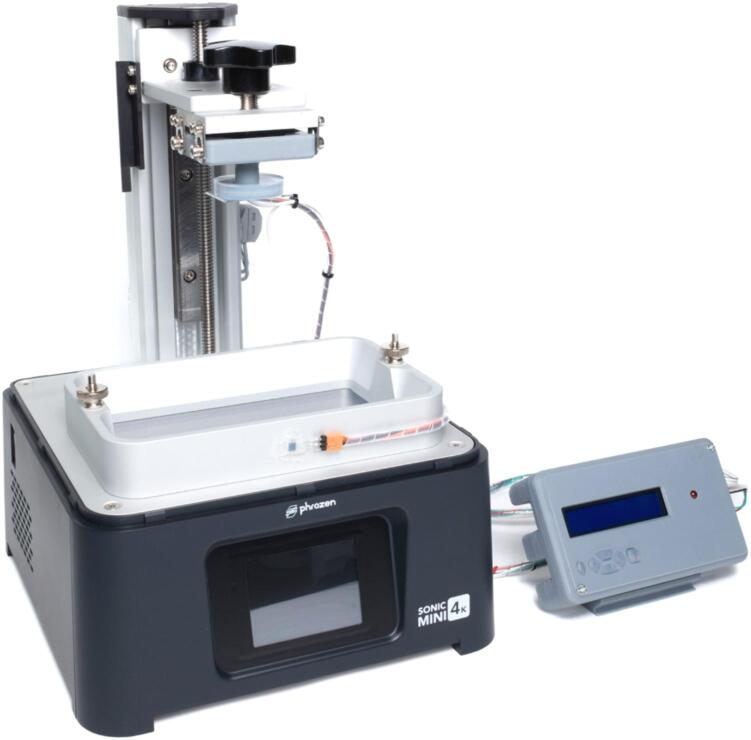
Modified mSLA bioprinter based on the Phrozen Sonic Mini 4 K.

With mSLAb, we present the easy conversion of an entry-level (€350) mSLA printer (Phrozen Sonic Mini 4 K) with off-the-shelf hardware and 3D printed components to a simple and low-cost open–source mSLA–based high-resolution 3D bioprinter. To enable the processing of ECM-based hydrogels and also cell-laden hydrogels, a temperature controller was integrated to ensure constant temperatures between 25 and 37 °C. In addition, a humidification process was implemented to prevent evaporation of liquid from the hydrogel. For easy sample handling and integrating the printed tissue construct in the workflow of cell cultivation and microscopic analysis, we implemented an innovative build platform for the attachment of glass substrates, see Fig. 1 and Fig. S1. All required 3D printed parts can be printed with the same printer that is being modified.

The commercially available Lumen X DLP bioprinter by Cellink offers similar pixel resolution (35 µm) and temperature control. However, it is not open-source and uses proprietary software, sacrificing customizability. The current model (Gen 3) is priced at over €30,000 per printer. To our knowledge, there are currently no relevant competitors with fully developed systems and support on the market. The startup TissueLabs offers a mSLA-based printer called TissueRay without temperature control, which restricts the range of compatible biomaterials to materials that are liquid at room temperature. Nevertheless, this system is marketed towards microfluidics and bioprinting, priced at over €20,000.

Conventional desktop SLA 3D printers are capable machines offering sub-50 µm resolution but lack the necessary features for bioprinting. Temperature control is essential for the use of the most prevalent biomaterials in order to ensure suitable viscosities during printing, as well as for the inclusion of living cells into the printing process. Moreover, the temperature control increases the flexibility and reproducibility of the printing process. Conventional 3D printers are equipped with large metal printing platforms, whereas glass substrates are generally preferred for biological applications. The use of individually sized transparent glass substrates in our setup increases flexibility and avoids unnecessary post-processing steps, thereby reducing the risk of corrupting the sample during handling. The prints can be analyzed using high-resolution optical microscopy without first transferring them to a suitable substrate. In addition, the modified printing platform allows for the use of hydrogel volumes as small as 100 µl, which is essential if expensive or custom-made hydrogels are used as 3D printing resin.

We have validated the system by printing macroscopic porous scaffolds as well as hollow channels as representative structures needed in tissue engineering. Printing macroscopic scaffolds demonstrates the ability to print large-scale hydrogel structures as tissue equivalents, e.g., for the 3D culture of cells for drug testing. Printing self-supporting hollow structures mimicking in–vivo vascularization is key to ensuring the viability of cells in larger-scale tissues. Combining these features may allow the generation of macroscopic tissue equivalents that can mature into functional tissue.

## 2 Hardware description

Under the premise of keeping the hurdle for setting up an mSLA-based bioprinting platform as low as possible for potential users, we chose as basis the Phrozen Sonic Mini 4 K Printer, a state-of-the-art consumer-level stereolithographic printer with 35 µm (XY) resolution and 405 nm lensed LED matrix illumination available for only €350. We kept the modifications and equipment needed at a minimum by implementing only a few new functions.

For mSLAb, the Phrozen Sonic Mini 4 K functionality has been supplemented by a temperature controller that allows for a constant temperature at approx. 35 °C, to ensure the protein-based resin's processability, humidification prevents resin's evaporation, and a redesign of the build platform enables direct printing on glass substrates, facilitating microscopic analysis of the printed 3D products. All modifications can be done in less than two working days using lab-made 3D printed parts, standard tools, and widely available standardized components. All 3D printed parts were printed with the same Phrozen Sonic Mini 4 K that was converted, eliminating the need for a separate 3D printer. The total cost of this bioprinter amounts to less than €500, which is a small fraction of the price of commercially available equivalents.

By publishing all necessary files for this mSLA–to–bioprinter conversion (mSLAb) as open-source files on publicly accessible repositories, we intend to enable the community to participate in the further development of the platform as well as to benefit from our own future revisions on an ongoing basis. We intend to present a sustainable concept for converting a commercial mSLA platform to a bioprinter that can be easily adapted to future mSLA printer generations and designs. Additionally, we anticipate mSLAb to be widely used in various research and teaching projects and aim for many customization options for the user. For this purpose, the software of the controller and the build platform can be readily adapted.

**Fig. 2:**
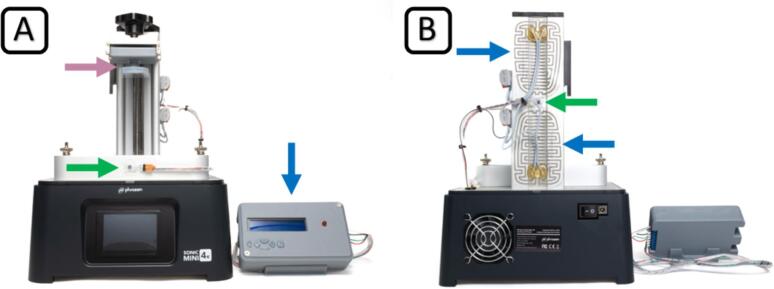
Modified mSLA bioprinter. Front view (A) with temperature controller (blue arrow), custom-designed build platform with glass substrate (purple arrow) and vat temperature sensor (green arrow). Rear view (B) with heating elements (blue arrows) and z-axis temperature sensor (green arrow). (For interpretation of the references to colour in this figure legend, the reader is referred to the web version of this article.)

For reliable temperature control, we took advantage of the high thermal capacity of the aluminum parts installed in the original printer and added resistive heating foils on the printer's z-axis (Fig. 2 (B, blue arrows)). An Arduino-based temperature controller (Fig. 2 (A, blue arrow)) allows the user to set a target temperature between 25–37 °C and heat the build chamber automatically and with minimal overshoot. Temperatures are measured near the heating elements at the printer's z-axis (Fig. 2 (B, green arrow)) and on the printer's material vat (Fig. 2 (A, green arrow)) and fed back to the controller. The controller includes a display that shows the current temperature of the vat as well as a status LED that shows the current state of the regulation (ref. to Chapter 6 for details).

This heating concept offers high temperature stability due to the high thermal masses. As a consequence, sample handling only leads to negligible deviations from the set temperature. Additionally, with this concept of temperature control, we can avoid using active fans that recirculate the air. This is crucial to prevent evaporation of the hydrogels used for bioprinting because hydrogels are liable to evaporation. Both their material and printing properties depend on their water content: If hydrogels lose water, the viscosity of the materials, as well as the relative content of additives such as photoinitiators, can increase, which in turn has a negative effect on the printing performance. In addition, the humidity in the printing chamber had to be increased to over 90 % to effectively prevent water evaporation from the hydrogels. For this purpose, we placed wetted sterile material with a high surface area within the vat (ref. to Chapter 6 for details). We also sealed the print chamber to protect the electronics from water vapor and to isolate the chamber from the airflow of the printer's integrated fan cooling the electronics.

**Fig. 3:**
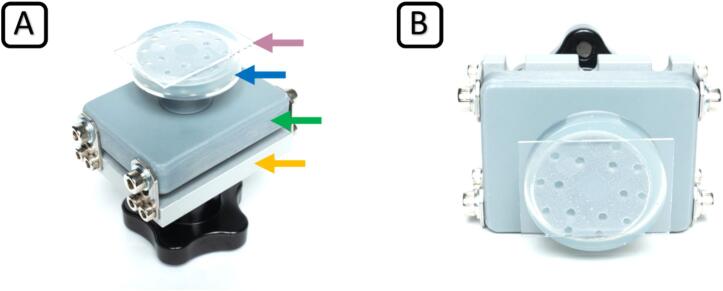
Custom-designed 3D-printed build platform (grey) with glass substrate (purple arrow). Silicone-encased build platform (blue arrow) and base plate (green arrow) are attached to the original attachment plate (orange arrow) in side view (A) and bottom view (B). (For interpretation of the references to colour in this figure legend, the reader is referred to the web version of this article.)

By adding a custom-designed 3D printed build platform (Fig. 3), we enable the use of glass substrates for printing. The bioprinted products can be handled conveniently in further downstream processes on these glass substrates, which neither interferes with microscopic analysis nor the cultivation of future cell-laden 3D constructs in, e.g., Petri dishes. Even more important: by using individually sized glass substrates, we could significantly reduce the amount of material required for printing, because protein-based resins used for bioprinting are typically scarce and expensive. Prints can be made with as little as 100 µl of hydrogel in this setup if desired. Due to the surface tension between the hydrogel, the plastic film covering the LCD, and the surrounding air, the hydrogel is confined in a small droplet between the glass substrate and the LCD cover, and the printing is executed within this droplet.

The redesigned build platform assembly consists of two pieces. First, a base plate (Fig. 3 (A, green arrow)) that connects to the original z-axis attachment plate (Fig. 3 (A, orange arrow)) and still supports the semi-automatic z-axis leveling implemented in the printer. Second, the exchangeable customizable build platform (Fig. 3 (A, blue arrow)), encased in transparent silicone, enables easy and secure attachment and removal of glass substrates by suction force.

• Compared to the well-known extrusion-based bioprinting, mSLAb allows for higher resolution, less printing time, and simultaneous printing of several products (multiplexing).
• Compatible with state-of-the-art bioprinting materials at physiological temperatures and 90 %+ relative humidity (rH).
• Generate complex 3D tissue equivalents for Tissue Engineering, biomedical research, drug testing, etc.
• At a total cost of only €500, the bioprinter offers a low-cost open-source alternative to commercially available systems.

## 3 Design files

All design files can be accessed in a repository under Mendeley Data.

### 3.1 Design files summary

**Table:** 

Design file name	File type	Open source license	Location of the file
Base Plate	CAD files	CC-BY-SA 4.0	https://doi.org/10.17632/kxt5sks9zs.1
Build Platform 30 mm	CAD files	CC-BY-SA 4.0	https://doi.org/10.17632/kxt5sks9zs.1
Cable Grommet	CAD files	CC-BY-SA 4.0	https://doi.org/10.17632/kxt5sks9zs.1
Case Bottom	CAD files	CC BY-SA 3.0	https://doi.org/10.17632/kxt5sks9zs.1
Case Stand	CAD files	CC BY-SA 4.0	https://doi.org/10.17632/kxt5sks9zs.1
Case Top	CAD files	CC BY-SA 3.0	https://doi.org/10.17632/kxt5sks9zs.1
Keypad Mold Bottom	CAD files	CC BY-SA 3.0	https://doi.org/10.17632/kxt5sks9zs.1
Keypad Mold Top	CAD files	CC BY-SA 3.0	https://doi.org/10.17632/kxt5sks9zs.1
Molding Stand	CAD files	CC-BY-SA 4.0	https://doi.org/10.17632/kxt5sks9zs.1
Printable Petri Dish	CAD files	CC-BY-SA 4.0	https://doi.org/10.17632/kxt5sks9zs.1
Sensor Seal Mold	CAD files	CC-BY-SA 4.0	https://doi.org/10.17632/kxt5sks9zs.1
mSLAb Code	Code	CC-BY-SA 4.0	https://doi.org/10.17632/kxt5sks9zs.1
Circuit Schematic	Figures	CC-BY-SA 4.0	https://doi.org/10.17632/kxt5sks9zs.1
Molding Process	Video	CC-BY-SA 4.0	https://doi.org/10.17632/kxt5sks9zs.1
Bioprinting Process	Video	CC-BY-SA 4.0	https://doi.org/10.17632/kxt5sks9zs.1

All CAD-type files consist of modifiable.step files,.stl files for slicing, and ready-to-print.ctb files.

• Keypad Mold Top and Keypad Mold Bottom are unmodified parts of “Mmintbox 1 Enclosure” by Vector_Mayhem under CC BY-SA 3.0.
• Case Top and Case Bottom are modified parts of “Mmintbox 1 Enclosure” by Vector_Mayhem under CC BY-SA 3.0. Modifications include holes for the LED, screw terminals, and magnets.
• mSLAb Code: Includes the code for the temperature controller and all necessary libraries to upload the code to the Arduino UNO.
• Circuit Schematic: High-resolution versions of the views shown in Fig. 5. Circuit schematic of the temperature control module (A) and component layout (B).
• Molding Process: Video showing the molding process of the platform, homing sensor seal, and button pad.
• Bioprinting Process: Video showing the bioprinting process to print, post-process, and image the two exemplary structures (Fig. 9 and Fig. 10).

## 4 Bill of materials

### 4.1 Bill of materials summary

**Table:** 

Designator	Component	Number	Cost per unit [€]	Total cost [€]	Source of materials	Material type
mSLA 3D-Printer	Phrozen Sonic Mini 4 K	1	349.99	349.99	3Djake	Non-specific
Temp. Controller	TSIC 506F TO92 Sensor	2	19.99	39.98	Conrad	Semiconductor
Temp. Controller	Heating pads (2x)	1	18.99	18.99	Conrad	Polymer/Metal
Temp. Controller	Arduino Uno	1	26.99	26.99	Conrad	Non-specific
Temp. Controller	LCD-Keypad Shield	1	9.99	9.99	Conrad	Non-specific
Temp. Controller	Wago connector	2	0.45	0.90	Conrad	Polymer/Metal
Temp. Controller	Perfboard	1	2.79	2.79	Conrad	Polymer/Metal
Temp. Controller	Heater Cable 2 x 0.75 mm^2^	1	1.49	1.49	Conrad	Polymer/Metal
Temp. Controller	Screw Terminal 1.50 mm^2^	4	0.20	0.80	Conrad	Polymer/Metal
Temp. Controller	Sensor Cable	2	1.46	2.92	Conrad	Polymer/Metal
Temp. Controller	12 V power supply	1	21.99	21.99	Conrad	Non-specific
Temp. Controller	Jumper wires	1	3.29	3.29	Conrad	Polymer/Metal
Temp. Controller	Resistor 220 Ω (10x)	1	0.35	0.35	Conrad	Metal
Temp. Controller	Resistor 2.2 kΩ (10x)	1	0.35	0.35	Conrad	Metal
Temp. Controller	Pins for sensor (10x)	1	0.18	0.18	Conrad	Polymer/Metal
Temp. Controller	Pins for perfboard (8x)	1	0.38	0.38	Conrad	Polymer/Metal
Temp. Controller	LED (red)	1	0.06	0.06	Conrad	Semiconductor
Temp. Controller	Spiral tubing 2/15 mm	2	0.35	0.70	Conrad	Polymer
Temp. Controller	Disc magnet 5 x 2 mm^2^	4	0.34	1.36	Magnet-Shop	Metal
Build Platform	Threaded inserts M4	8	0.39	3.12	Conrad	Metal
Build Platform	M4 x 10 screw	5	0.14	0.70	Conrad	Metal
Build Platform	M4 x 30 screw	3	0.18	0.54	Conrad	Metal
Build Platform	M4 nut	3	0.06	0.18	Conrad	Metal
Build Platform	3 x 12 mm screw (100x)	1	2.01	2.01	Conrad	Metal
Build Platform	2-Part Silicone SF45	1	7.75	7.75	Silikonfabrik	Polymer
		TOTAL		497.80		

## 5 Build instructions

The mSLAb concept is based on using custom-made printed parts to modify the Phrozen Sonic Mini 4 K. All parts can be 3D printed using standard purchasable (synthetic) resins (i.e., Phrozen Aqua Grey 4 K) using the same printer that will be modified. All STL files have been prepared accordingly. In addition, ready-to-use sliced print jobs for the Phrozen Aqua Grey 4 K resin are available. Surfaces of the 3D printed parts that will be in direct contact with silicone have to be post-cured after printing by additional UV exposure for 1 h or heat treatment (120 °C for 4 h) [31], [32]. Alternatively, all parts can be printed using FDM 3D printers and standard PLA filaments.

### 5.1 Assembly of the build platform

The first step is to assemble the new build platform and molding stand to encase the platform in silicone.

**Fig. 4:**
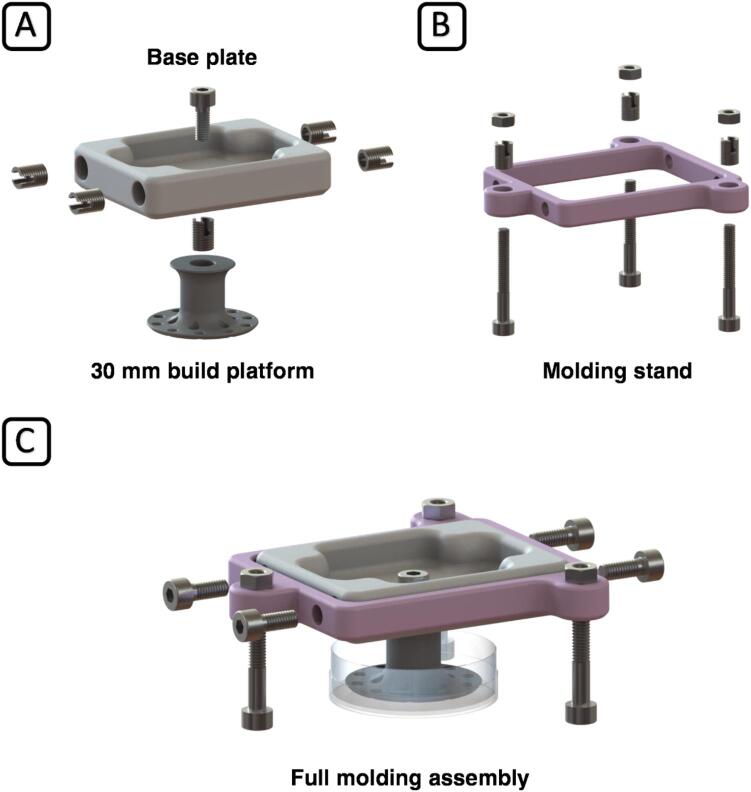
Detailed overview of the build platform (A), molding stand (B), and the complete molding assembly (C), including the positions of the thread inserts and screws.

Install ENSAT nut inserts into the base plate (4x), build platform (1x), and molding stand (4x), as shown in Fig. 4 (A, B). Use a flat-head screwdriver (size 2) and pay special attention to the perpendicular alignment of the insert when screwing it in slowly. Mount the build platform onto the base plate and the base plate to the molding stand using M4 x 10 screws and an M3 hex key. Suspend the platform in a suitable (30 – 35 mm) plastic Petri dish using the three M4 x 30 screws to set a gap of 2 mm between the bottom of the platform and the inside surface of the Petri dish, as shown in Fig. 4 (C). Alternatively, a Petri dish can be 3D printed using the printable Petri dish file included in the design files. Make sure to level the surface of the platform in relation to the Petri dish and secure it with the M4 nuts. The platform is now ready for the molding process described in Chapter 5.2. After molding, exchange the original aluminum build plate of the Phrozen Sonic Mini 4 K for the custom silicone-encased build platform.

### 5.2 Fabrication of silicone parts

This Chapter describes the fabrication of the three custom silicone parts necessary for the conversion (silicone-coated platform, sensor seal, and keypad for the controller). We have provided a video demonstration of the molding process in the data repository.

The silicone parts are molded using a single SF45 self-mixing cartridge − an affordable two-part silicone that will cure at room temperature (RTV). Alternatively, PDMS (Dow Corning Sylgard 184) or another suitable silicone with comparable properties (45 Shore A hardness) may be used.

Always fill the molds carefully from the bottom up to avoid air pockets. Fill the sensor seal mold up to the outer rim. Fill both halves of the keypad mold and assemble the mold. Fill the Petri dish up to 5 mm and slowly suspend the platform assembly in the center of the Petri dish. Leave to polymerize for at least 2 h at room temperature (preferably overnight). Remove the finished keypad by lifting off the lid of the mold and carefully pry out the pad. The sensor seal mold has defined weak points, enabling easy seal removal. Break away the outer rim and slide out the seal from the remaining post on the base plate. Unscrew the base plate of the build platform from the molding stand. The silicone-encased build platform can be removed by breaking the Petri dish with a wire cutter. Do so by carefully cutting into the plastic above the silicone on two opposing sides to relieve the tension in the dish. Clean the silicone parts with isopropyl alcohol (IPA) before use.

### 5.3 Assembly of the temperature controller

This Chapter describes the assembly of the temperature controller.

**Fig. 5:**
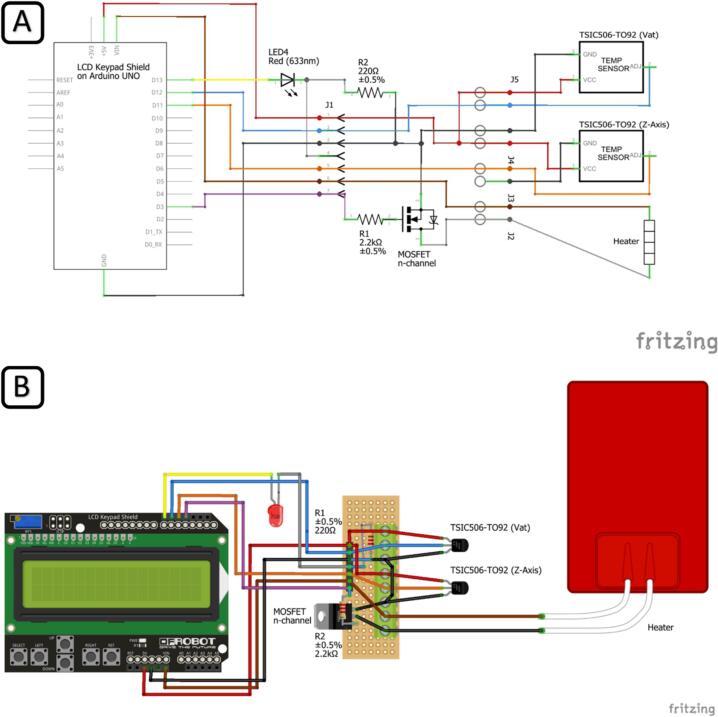
Circuit schematic of the temperature control module (A) and component layout (B).

**Fig. 6:**
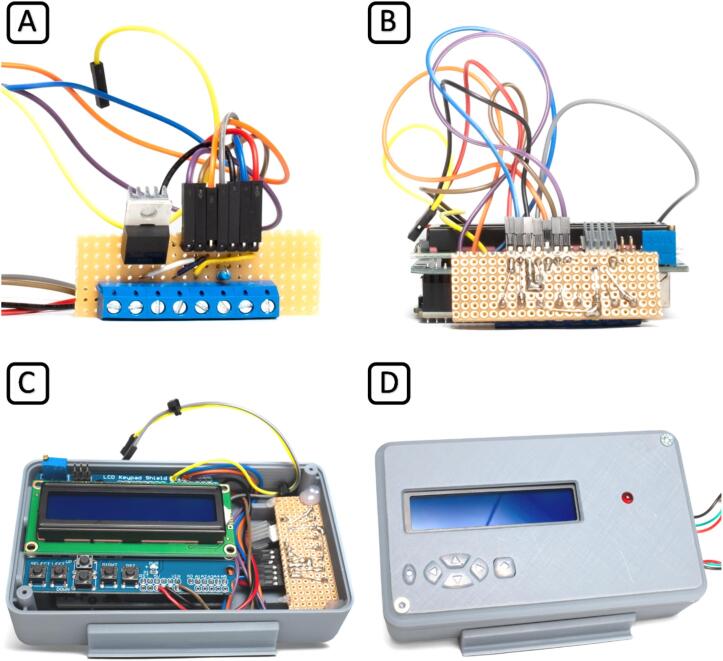
Temperature controller. The exemplary component layout on the perfboard front (A) and back (B). Arduino and perfboard, placed within the 3D-printed case (C) and finished temperature controller (D).

First, assemble and solder the perfboard and plug the keypad shield on top of the Arduino Uno according to Fig. 5, showing the circuit schematic (A) and component layout (B) created with the software Fritzing [33]. We have also provided photos of the layout as well as an overview of the positions of the components within the controller case in Fig. 6. Now connect the perfboard to the pins on the keypad shield. We recommend using detachable connectors to connect the perfboard with the Arduino, but cables may be soldered directly between the components instead. Place the LED into the case top cover and fix it with glue if necessary. For the 3D printable magnetic stand for the temperature controller case, two magnets (5 x 2 mm) must be glued into the provided blind holes in the stand and the case. Place electronics into the case bottom and fix them with hot glue if necessary. Insert the silicone keypad into the case top, close the case, and screw it tight using two 3 x 12 mm screws.

### 5.4 Printer modifications

This Chapter describes the modifications that are made directly on the printer.

**Fig. 7:**
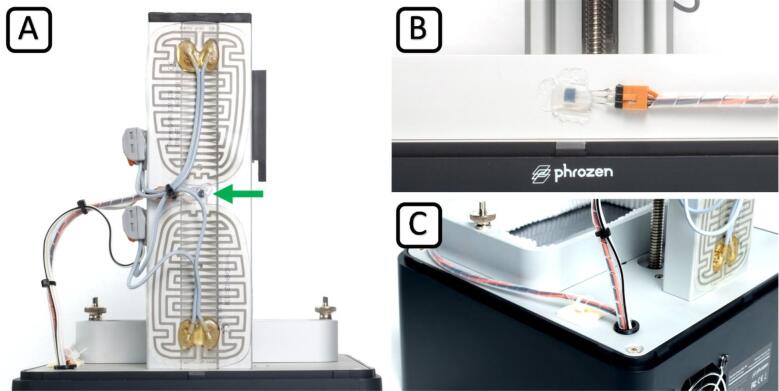
Close-up views of the bioprinter modification. Placement of the heating pads, z-axis temperature sensor (green arrow) and wiring routing (A), vat temperature sensor placement (B), and cable grommet (C). (For interpretation of the references to colour in this figure legend, the reader is referred to the web version of this article.)

Disconnect the 3D printer from the power supply at least 30 min before starting the modification. Lay the 3D printer on its back and remove the bottom plate by removing the four M4 screws with an M3 hex driver and disconnecting the LED array connector from the mainboard. Carefully set the plate aside and take care not to touch the lens array – cover it to protect it from dust. Place the printer back upright and drill the 10 mm hole for the wiring at a distance of approximately 25 mm from both sides of the corner. Use a suitable drill at a moderate speed and vacuum the metal swarf. Deburr the hole, remove any remaining swarf, and apply the cable grommet. Lay the printer on its back and remove the three screws of the mainboard. Carefully move the mainboard just far enough to slip the sensor seal over the homing sensor. Refit the mainboard and screw it tight. Check the fit of the sensor seal by looking into the cutout in the aluminum plate from above. The seal must not impair the function of the sensor. Apply the adhesive heating pads to the back of the z-axis of the printer (see Fig. 7 (A)).

We advise soldering pin terminals to the sensors to ensure a firmer fit with the removable cable connectors, see Fig. 7 (B). Glue the sensors on the z-axis and resin vat with a thermal adhesive (Fig. 7 (A, B)). Apply a thin coat of hot glue to the sensor for additional stability and insulation. Insert the cables for the sensors and heating pads through the grommet and route them to the outermost pre-cut hole in the baseplate. We encourage using spiral cable wrap, self-adhesive pads, and cable ties, see Fig. 7 (A, C). Cable ties on both sides of the grommet function as a strain relief. Reconnect the LED array and refit the baseplate to the printer by tightening all four screws. Connect the cables from the printer to the temperature controller to finish the electronic installation.

Download and install Visual Studio Code on a PC using the default settings. Start the application and install the extension PlatformIO via the Extensions tab. After successful installation, load the “mSLAb_Code” folder into the Workspace of PlatformIO. Connect the Arduino Uno microcontroller to the PC via USB and upload the code by pressing the upload button in the lower left corner. Upon finishing the upload, the terminal will display “success,” and the temperature controller will be ready for use. Detailed information about the temperature regulation is provided in the comments in the code.

## 6 Operation instructions

We have provided a video of an exemplary print procedure in the data repository, including the prints demonstrated in Chapter 7. Additionally, the process is illustrated in a flow chart in the Supporting Information (Fig. S5).

To start printing, turn on the temperature controller by connecting the power supply (12 V at least 24 W) and turn on the printer. The LCD will initialize, and the LED will turn on. The LED ceases to be on constantly but flashes as soon as the temperature is within 2 °C of the set temperature and turns off as soon as it is within 0.2 °C of the set temperature. The temperature can be adjusted in 1 °C increments using the up and down arrows and 0.1 °C increments using the left and right keys. Set the desired temperature – we recommend 35 °C to reduce the viscosity of the material and ensure cell compatibility when using cell-laden materials. The exothermic nature of the polymerization may introduce additional heat, which is why we recommend a slightly lower starting temperature than physiological conditions of 37 °C [34]. Caution: Temperatures near the heating pads may reach up to 55 °C.

Remove the printer's cover only for sample handling and cleaning procedures to ensure good temperature distribution. To minimize evaporation and extend the maximum possible printing time, we recommend increasing the air humidity by placing a wetted high surface material into the vat, like a sponge. We use two disposable surgical head caps (Klipphauben Medium PP-Spinnvlies; Ampri GmbH, Luhe, DE) cut at 2/3 length, soaked in ddH_2_O, and placed along the sides of the vat. This method typically yields a relative humidity of over 90 %, reducing evaporation loss and thus supporting prints with hydrogels for up to multiple hours.

Remove the printing platform and place a freshly cleaned glass substrate (e.g., halved standard microscopy slides) on the cleaned silicone surface. Use IPA and lint-free cloths to clean the glass and silicone surfaces. If there has been a change in substrate or platform combination, the platform will need to be re-calibrated before starting the print job. Note that the calibration must be performed with the glass substrate attached. The z-axis calibration procedure is analogous to regular resin printing and is a guided feature implemented into the Phrozen Sonic Mini 4 K printer software; detailed instructions are also available online. We have kept the same four-screw setup and approximate measurements to ensure compatibility with the semi-automated leveling procedure of the printer manufacturer.

Preheat the hydrogel to 37 °C – we used a lab-made GelMA-based hydrogel for the exemplary prints. Prepare the print job using a compatible slicing software – we recommend Lychee slicer by Mango3D. We advise the addition of a circular raft (Scale 100 %, Thickness 0.4 mm) to improve adhesion. Note the automatically calculated net material volume needed for the print. Pipet 2–3 times the calculated net print volume of preheated hydrogel into the center of the resin vat. The exact volume needed to ensure the last printed layer is permanently suspended in unpolymerized material during the print procedure will depend on a multitude of factors, including the surface area/volume/geometry and the substrate size due to capillary forces. Adding more material during the printing process may be necessary for larger print volumes.

Close the printer cover, turn on the printer, select the print job, and start the print. Once the print is finished, remove the platform and then remove the glass substrate. The print should be washed by placing it in a container filled with prewarmed solvent (typically ddH_2_0 or PBS, depending on the application) to remove excess unpolymerized hydrogel. Use a wash bottle or syringe to wash out porous structures and channels actively. The prints may be cured by additional exposure to light (405 nm) to enhance crosslinking and thus increase stability and reduce swelling. Incubate or place on a heating plate at 37 °C and change the solvent at least three times during the first 24 h to improve transparency.

## 7 Validation and characterization

The conversion of the Phrozen mSLA printer for bioprinting applications does not affect the precision, resolution, and tolerances (=basic mechanical parameters) since the mechanics remain unchanged (see Table 1).

**Table 1:** Phrozen Sonic Mini 4 K technical specifications.

Parameter	Set value
Layer height (z res)	10 – 300 µm
Pixel Size (xy res)	35 µm
Light source	ParaLED Matrix 2.0 (lensed LED matrix/array)
LED Wavelength	405 nm
Printing Display	6.1″ monochrome 4 K LCD, 3840 x 2160 px
Printer size	250 x 250 x 330 mm^3^
Printer weight	5 kg
Max print volume	134 x 75 x 130 mm^3^

The final resolution or minimum feature size achieved in bioprinting depends on a variety of factors, including the material used (such as the capability to be crosslinked to a solid, polymerization/absorption characteristics, photoinitiator system, and viscosity) and the printing parameters chosen (such as exposure, z-layer height, movement speed, and print orientation).

For the prints presented in Fig. 9 and Fig. 10, we used GelMA, which was synthesized in our lab at a final concentration of 15 % (w/v) supplemented with 0.6 % (w/v) Lithium phenyl-2,4,6-trimethylbenzoylphosphinate (SKU: VLLP00010010, CELLINK) and 0.06 % (w/v) Quinoline Yellow (SKU: 277340250, Thermo Fisher Scientific) for crosslinking at 405 nm. The synthesis was based on the RO–12 h–L GelMA variant published by Kumar et al. [35]. Commercially available GelMA is also well suited for this purpose.

In mSLA-based printing, the material has to be liquid to enable the layer-by-layer polymerization process. Printing is further improved by reduced viscosity, because the process involves up and down movement of the build platform after each consecutive layer is cured, to allow fresh resin to flow into the gap between the newly cured layer and the bottom of the vat. A lower-viscosity material will flow more easily and evenly, creating a consistent thin layer for the next exposure to light. This also helps to avoid defects in the constructs and cell damage caused by shear forces or air bubbles. GelMA exists in a gelatinous state at room temperature, making it too viscous to be processed in the mSLA process. However, upon heating the material to 35 °C, it undergoes a phase transition to a liquid state with significantly reduced viscosity, making it suitable for printing. Therefore, in order to successfully print with GelMA, it is essential to modify the mSLA printer and include a temperature control for the printing chamber. Additionally, this feature offers the possibility for future printing of hydrogels laden with living mammalian cells, which require an ambient temperature of 37 °C. However, the increase in temperature favors the evaporation of water from the GelMA, thus increasing its protein concentration. For consistent printing quality, the material properties should remain constant, and evaporation should be avoided by humidification.

In this section, we, therefore, aim to validate the system concerning temperature stability and humidification. We show two representative products printed directly onto a glass substrate using the silicone-encased build platform.

### 7.1 Validation of temperature control and humidification

**Fig. 8:**
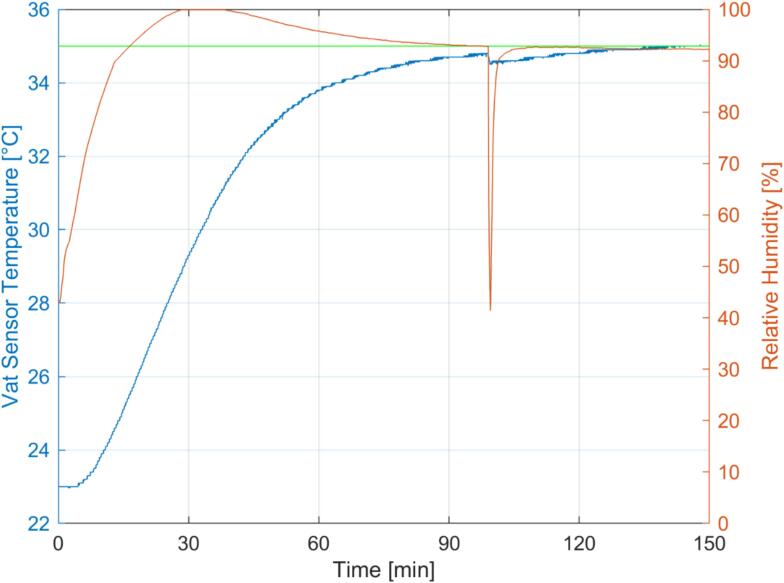
Exemplary temperature and humidity curve during the start-up of the bioprinter. Vat Sensor Temperature (blue), Relative Humidity (orange), and Temperature Set Value (green). The printer's top cover was removed for sample handling at 99 min. (For interpretation of the references to colour in this figure legend, the reader is referred to the web version of this article.)

The mSLAb printer is equipped with wetted disposable surgical head caps, and the target temperature is set to 35 °C (ref. to Chapter 6) to prepare for the printing process. Fig. 8 shows the typical curve of temperature and humidity measured in the printing area. After about 80 min, the target temperature is almost reached, with a deviation of only about 0.5 °C. The humidity rises much faster, reaches nearly 100 % after 30 min, and stabilizes at 93 %, which is sufficient to prevent evaporation. After 99 min, the cover of the printer was removed for 30 s to simulate sample handling. Note that light exposure causes a temperature increase within the printing material (due to the exothermic nature of the polymerization reaction), which is why the temperature in the printing chamber is set to 35 °C, although a printing temperature of 37 °C is aimed for. The graph shows the negligible temporary impact of sample handling on the temperature curve. Both temperature and humidity remain almost constant during printing.

Please note: We validated the temperature control with the provided build platform (30 mm) for glass substrates. If a larger build area is to be used (i.e., a larger build platform), the temperature distribution and stability in the corresponding printing area should be validated thoroughly.

### 7.2 Representative prints / validation of printing performance

**Fig. 9:**
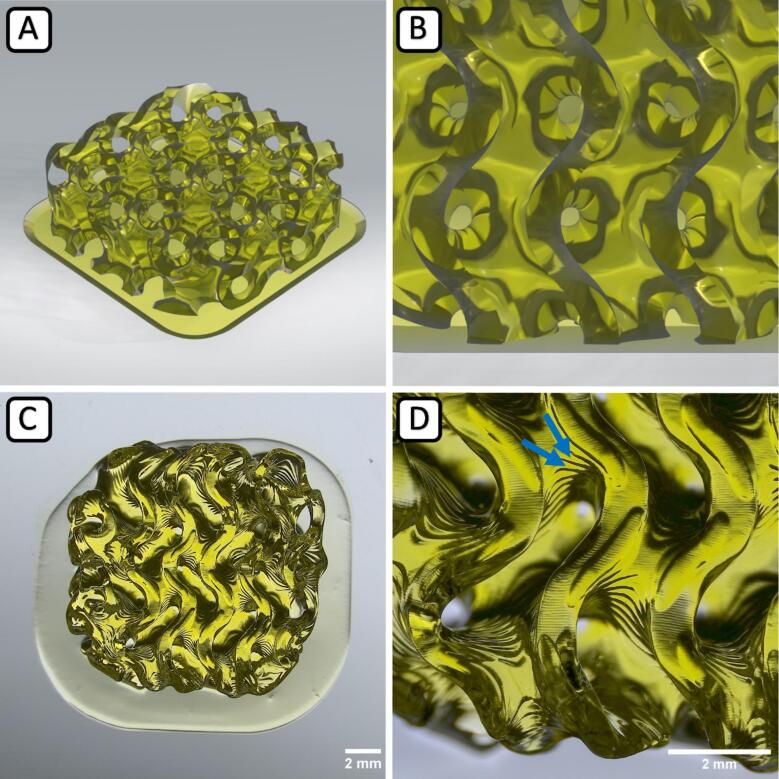
Macroscopic Gyroid scaffold. Rendered image (CAD) in isometric view (A) and close-up (B). Microscopy image of the printed scaffold using GelMA hydrogel in overview (C) and close-up (D) with visible 50 µm z–layers (D, blue arrows). (For interpretation of the references to colour in this figure legend, the reader is referred to the web version of this article.)

**Fig. 10:**
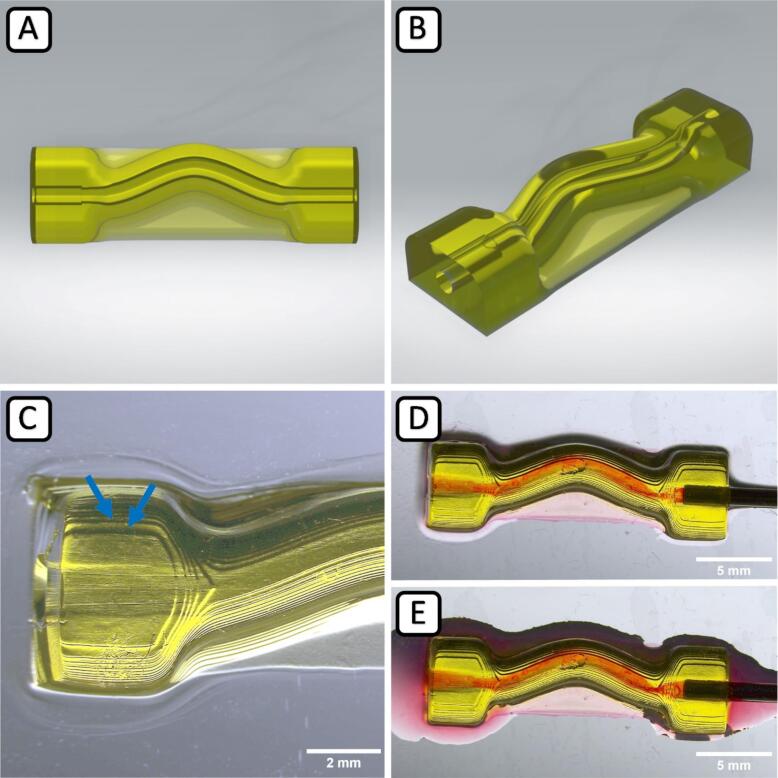
Perfusable channel construct. Rendered image (CAD) in overview (A) and isometric view (B). Microscopy image of the printed channel construct using GelMA hydrogel in overview (C) with visible 50 µm z–layers (C, blue arrows) and during perfusion (D, E). (For interpretation of the references to colour in this figure legend, the reader is referred to the web version of this article.)

To validate the printing process of gelatinous hydrogels with mSLAb and to judge the quality of the printed sample, we chose two different test structures: A gyroid cube structure with dimensions of about 10 x 10 x 3 mm^3^ possessing a high porosity resulting from channels communicating with each other (Fig. 9). This structure allows for the distribution of growth medium and oxygen within the printed construct. This is essential for the cultivation and survival of living cells that may be seeded on the construct subsequently or added to the GelMA and integrated directly into the printing process, which is the next step of development we aim for. Such porous geometries are frequently used in 3D cell culture. The second structure is a hollow tube that can be perfused with liquid or growth medium and mimics a simplified model for vascular structures in Tissue Engineering (Fig. 10).

Both structures were printed from GelMA hydrogel as the material, as it is the most prevalent photocrosslinkable polymer in bioprinting. Samples were printed at 50 µm z-resolution at 35 °C. After the initial heat–up of the printer, the cover was removed to apply 800 µl of prewarmed (37 °C) GelMA to the build platform, and the printing process was started after reinstalling the cover. The data repository provides a video that includes the print procedure, post-processing, and imaging of the two samples shown here.

Fig. 9 shows a 3D rendering of the CAD file of the gyroid cube (A) and a close-up of the surface (B) to illustrate its porosity. The corresponding printed construct of GelMA is shown in (C) in total and in (D) as a surface close-up. The dimensions and the geometry of the printed construct correspond almost exactly to the specification of the original design, proving the high shape fidelity of GelMA printing with mSLAb. The layer-by-layer construction and the uniformity of the individual layers can be seen particularly well in the close-up (D), where the 50 µm z–steps from layer to layer are clearly visible (D, blue arrows).

Fig. 10 (A, B) depicts 3D renderings of the perfusable tube construct. Also, in this case, the printed construct's geometry hardly deviates from the original design (C-E). Again, the close-up in (C) shows the 50 µm z–layering of the wall structure surrounding the hollow channel (C, blue arrows). The mechanical stability of this tube construct is high enough to allow the insertion of a cannula and the perfusion with liquid, proofing that the inserted channel is open and permeable (D).

Additional 3D printed items as well as the corresponding parameters are displayed in the Supporting Information (Fig. S2 – S4).

### CRediT authorship contribution statement

**Benedikt K. Kaufmann:** Conceptualization, Data curation, Investigation, Methodology, Visualization, Writing – original draft. **Matthias Rudolph:** Investigation, Methodology, Software, Validation. **Markus Pechtl:** Investigation, Software, Validation. **Geronimo Wildenburg:** Investigation, Validation. **Oliver Hayden:** Supervision, Writing – review & editing. **Hauke Clausen-Schaumann:** Funding acquisition, Project administration, Writing – review & editing. **Stefanie Sudhop:** Conceptualization, Supervision, Writing – review & editing.

## Declaration of competing interest

The authors declare that they have no known competing financial interests or personal relationships that could have appeared to influence the work reported in this paper.
