# Supplementary material for: mSLAb – An open-source masked stereolithography (mSLA) bioprinter
Source: HardwareX. 2024 Jun 10;19:e00543. doi: 10.1016/j.ohx.2024.e00543 (PMC11234000; doi:10.1016/j.ohx.2024.e00543)
Supplement: Supplementary Data 1 [file mmc1.pdf]

SUPPORTING INFORMATION

mSLAb – an open-source masked stereolithography (mSLA) bioprinter

Authors

Benedikt K. Kaufmann[123]*, Matthias Rudolph[1], Markus Pechtl[1], Geronimo Wildenburg[1], Oliver Hayden[3], Hauke Clausen-Schaumann[12], Stefanie Sudhop[12]*

Affiliations

[1]Center for Applied Tissue Engineering and Regenerative Medicine, Munich University of Applied Sciences, 80335 Munich, Germany

[2]Center for NanoScience – CeNS, Ludwig Maximilian University of Munich, 80539 Munich, Germany

[3]Heinz-Nixdorf-Chair of Biomedical Electronics, School of Computation, Information and Technology & Munich Institute of Biomedical Engineering, Technical University of Munich, TranslaTUM, Einsteinstraße 25, 81675 Munich, Germany

Stefanie.Sudhop@hm.edu; Benedikt.Kaufmann@hm.edu

This file provides a figure summarizing the modifications made to the device, additional experiments to validate the mSLAb 3D printer, exemplary print settings, and a flowchart for the printer's operation.

The PDF file includes:

1. Modifications Overview
2. Additional prints
3. Exemplary print settings
4. mSLAb operation flowchart

----

1. Modifications Overview

Fig. S1 shows an overview of all major modifications made to the Phrozen Sonic 4K 3D printer. The build platform (A) was redesigned for exchangeable glass substrates, enabling microscopic analysis of the printed constructs without sample manipulation, and is optimized for reduced material consumption. Light-based bioprinting with protein-based biomaterials like GelMA requires temperature control. We added resistive heating pads to the aluminum back of the z-axis (D) and temperature sensors to monitor the temperature near the heating pads (D, green arrow) and the material vat (C). Necessary wiring was routed through a hole in the base plate (B) so the original printer lid could be used. The temperature controller is a separate module based on an Arduino Uno microcontroller combined with an LCD keypad shield as a user interface (E). The combination of these modifications enables the use of protein-based photopolymers (e.g., GelMA) for 3D tissue engineering.

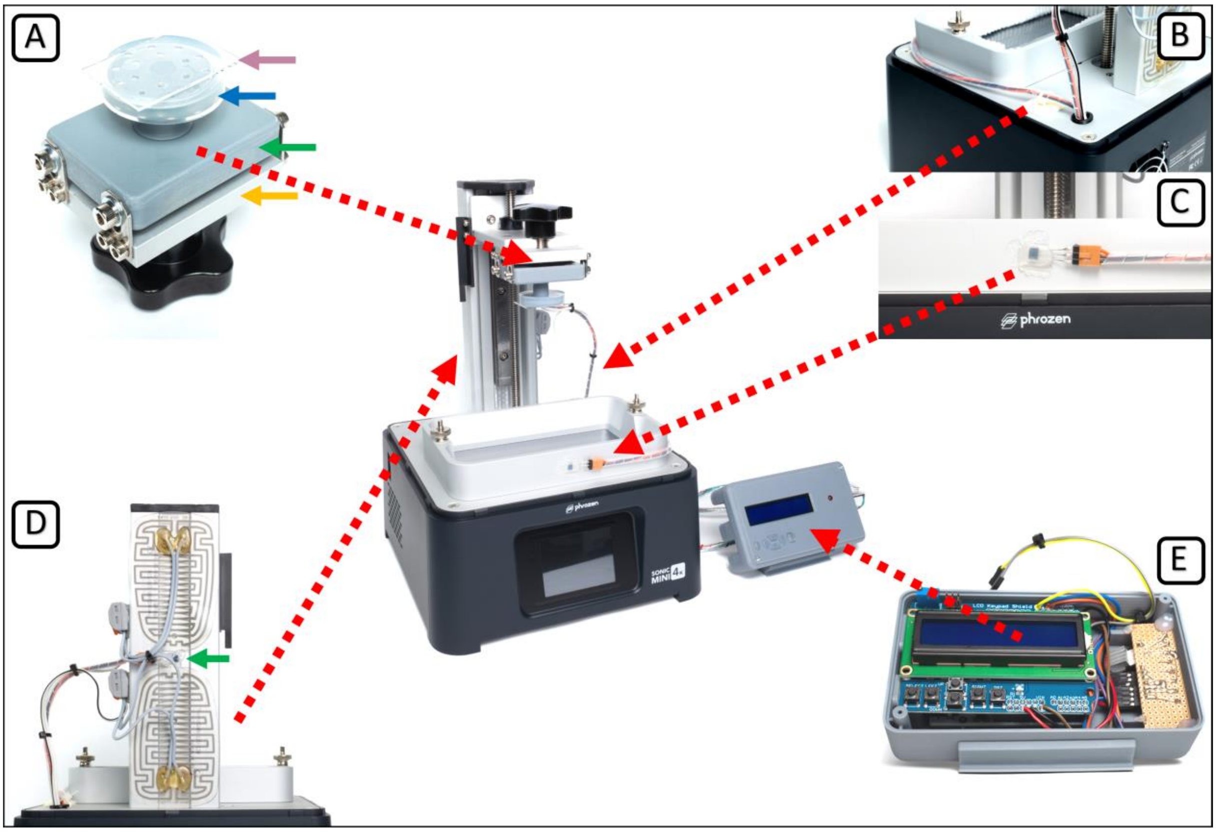

Fig. S1: mSLA bioprinter modifications overview. Custom-designed build platform (A) with exchangeable glass substrate (purple arrow). The silicone-encased build platform (blue arrow) and base plate (green arrow) are attached to the original attachment plate (orange arrow). Temperature control is achieved by combining a microcontroller-based temperature controller with an LCD keypad user interface (E) with resistive heating pads on the back of the aluminum z-axis (D). Sensors to monitor temperatures were placed on the z-axis between the heating pads (D, green arrow) and the front of the material vat (C). Cables for sensors and heaters are neatly routed via a hole in the printer's base plate (B).

2. Additional prints

Here, we demonstrate the versatility of the platform and the capability to print small individual lines and larger complex structures by printing additional models with a second protein-based material.

For the prints presented in Fig. S2 and Fig. S3, we used GM10, which was synthesized in our lab at a final concentration of 20% (w/v) supplemented with 0.6% (w/v) Lithium phenyl-2,4,6-trimethylbenzoylphosphinate (SKU: VLLP00010010, CELLINK) and 0.06% (w/v) Quinoline Yellow (SKU: 277340250, Thermo Fisher Scientific). GM10 is a proteinaceous resin with a high degree of functionalization, made using gelatine of a different species (bovine) [1].

Fig. S2 shows an array of sinusoidal lines with a width of less than 100 µm on an adhesion platform (15 x 15 x 0,5 mm³). The array of nine 3D sinusoidal lines was printed without defects, and the lines have an average width of less than 2 pixels, which is nearly independent of orientation. Note that the individual pixels are visible within the flat adhesion platform.

The average width of the sinusoidal lines measured in this print was 52.38 µm. The width was measured using ImageJ (Fiji Distribution) and the InteredgeDistance Macro. In brief, the macro uses two splines drawn along the edges of the lines in the microscopy image (Fig. S2 D), and the distance between the edges was measured in 97 equidistant locations along the splines (mean: 52.38 µm, standard deviation: 5.69 µm).

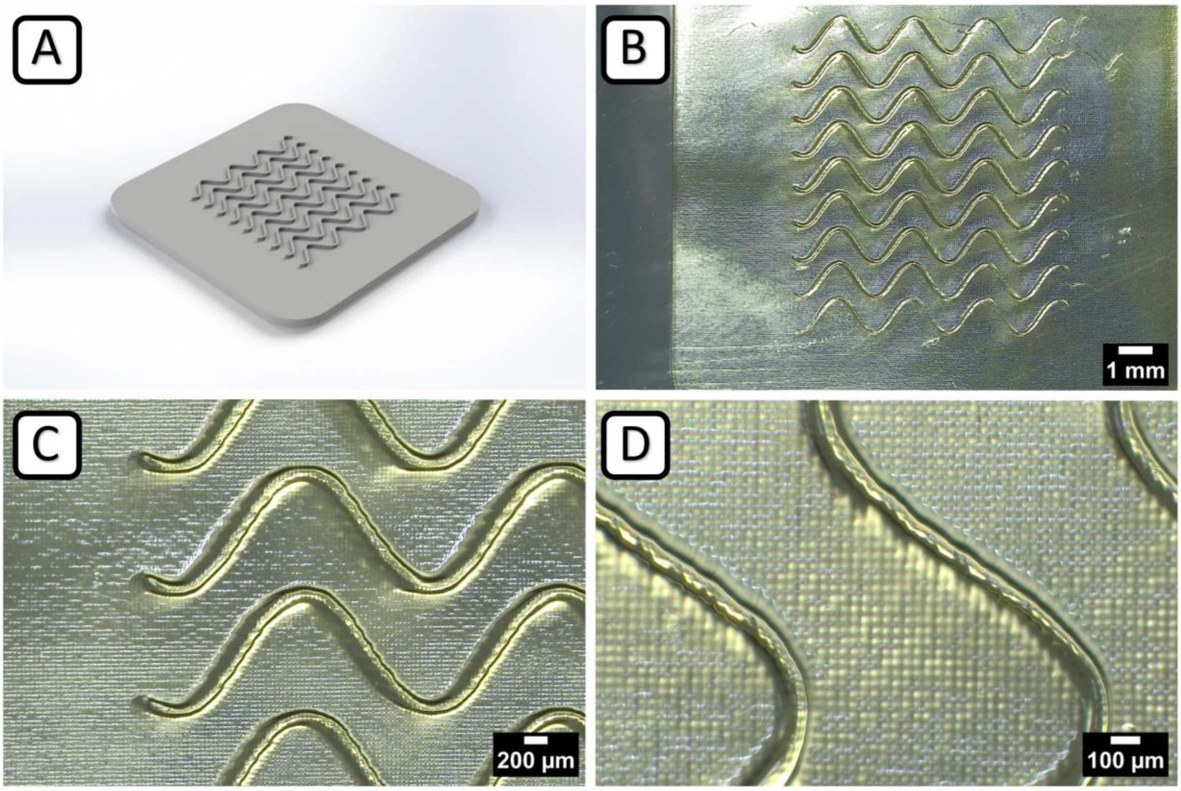

Fig. S2: Sinusoidal Line Array 3D printed on mSLAb with GM10 biomaterial. Rendered image (CAD) in isometric view (A). Microscopy images with different degrees of magnification of the structures printed on the mSLAb with GM10 material (B-D). Note the fully formed 3D sinusoidal lines with a mean 52.38 µm width. The pixel size is visible inside the flat adhesion platform, especially at higher magnifications (C-D).

Fig. S3 shows a larger-scale scaffold design consisting of square profile beams (500 x 500 µm) stacked orthogonally to the size of 10 x 10 x 10 mm³ on an adhesion platform (15 x 15 mm²). Fig. S3 (C-F) show the high wetting properties of the scaffold structure. This is ideal for subsequent cell seeding for tissue engineering and diffusion of nutrients throughout the structure during cultivation. Fig. S3 (G-H) show a top view of the scaffold with alternating beams and hollow spaces clearly visible. We varied the focus depth from the top (G) to the bottom (H) to show the three-dimensionality of this comparably high structure and the uniformity of the beams throughout the construct.

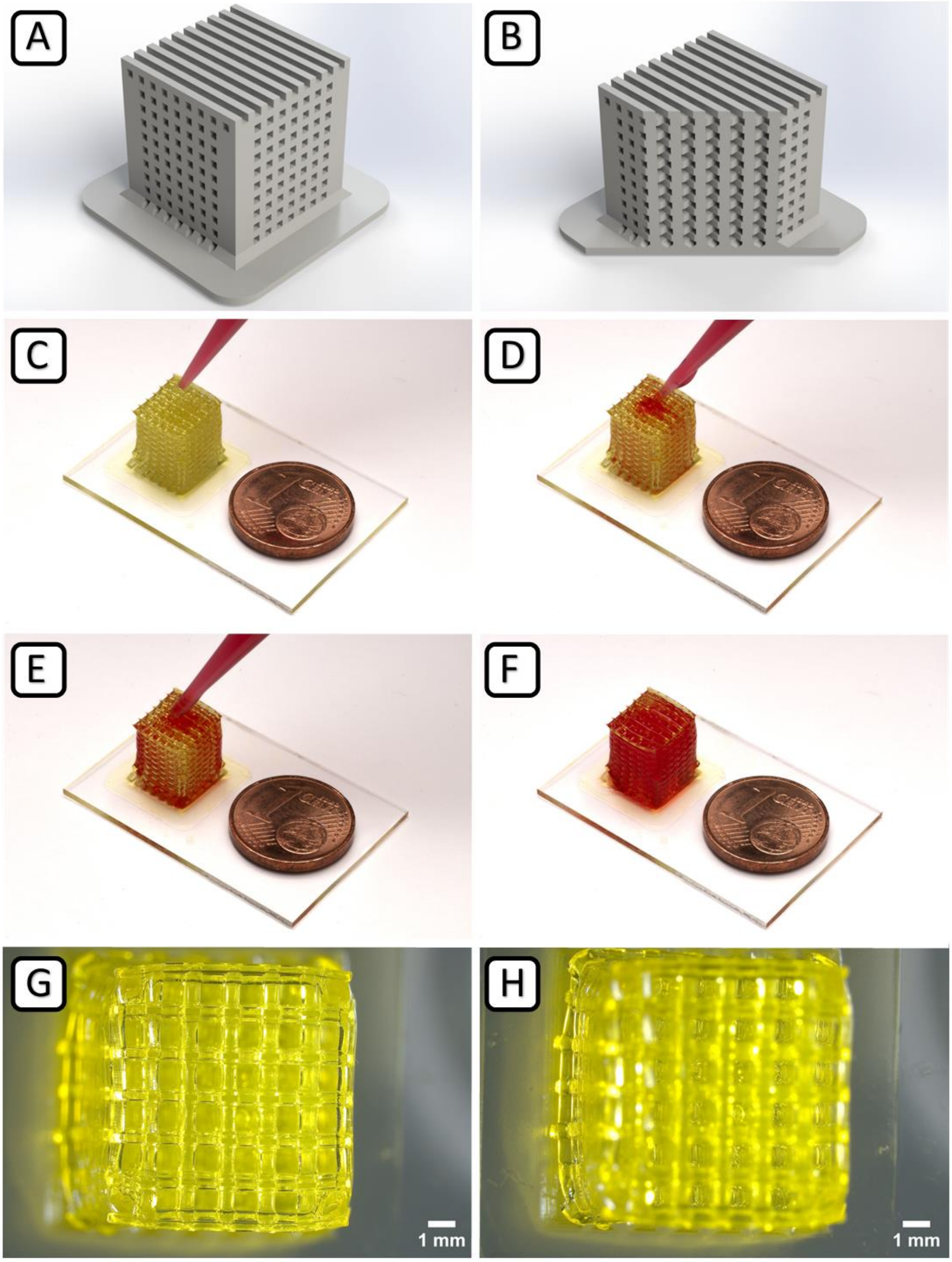

Fig. S3: Complex orthogonal line scaffold (10 x 10 x 10 mm³) 3D printed on mSLAb with GM10 biomaterial. Rendered image (CAD) in isometric view (A) and cut view to visualize internal features (B). Photography images demonstrate the internal structure's excellent wetting properties, which are essential for subsequent cell seeding and cultivation in tissue engineering (C-F). Microscopy images showing the alternating beams and hollow spaces with varied focus on the top (G) and bottom (H) layers demonstrate that the lattice structure has been realized throughout the entire construct.

3. Exemplary print setting

Fig. S4 shows exemplary print settings for printing with GelMA on the mSLAb system. These exact settings were used to print the complex orthogonal line scaffold in Fig. S3. "Burn In" layers with long exposure times facilitate good adhesion of printed structures to the substrate. "Normal" layer settings apply after the number of "Burn In" and "Transition" layers are completed. "Transition" layers provide a linear gradient of varied exposure time to bridge the gap between the settings for "Burn In" and "Normal" layers and reduce possible tension between layers printed with noticeably different exposure times, which otherwise may lead to delamination of layers and failed prints. We chose the number of "Burn In" (6) and "Transition" (4) layers in a 60%/40% fashion to amount to the number of layers needed to print the adhesion platform (500 µm thickness) designed into the bottom of all models printed with GelMA/GM10 (see Fig. S2 (A) and Fig. S3 (A)). The desired structure (i.e., sinusoidal lines or orthogonal line scaffold) is then polymerized exclusively and homogenously with the settings for "Normal" layers.

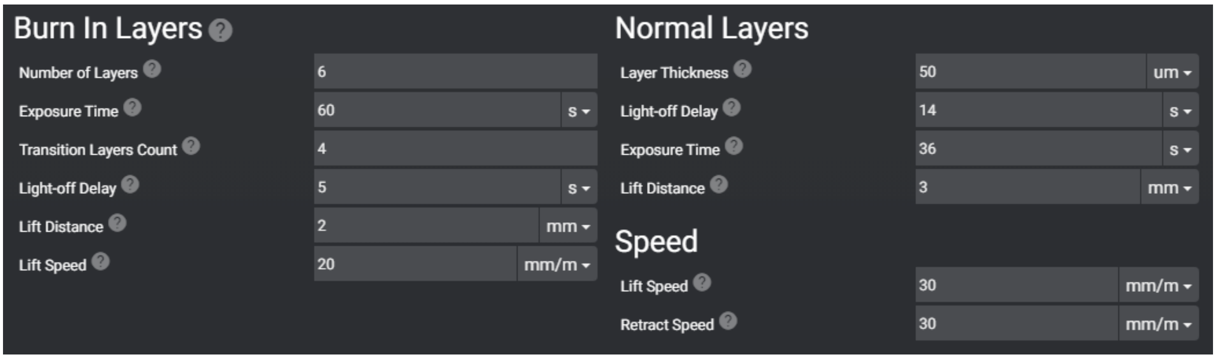

Fig. S4: Screenshot of exemplary print settings in Lyche Slicer software. These exact settings were used for printing the complex orthogonal line scaffold in Fig. S3 and should prove helpful as initial values for optimization when printing with GelMA. "Burn In" layer settings provide extended exposure to facilitate good adhesion of the first layers to the substrate, while "Normal" layer settings provide optimized exposure for finely detailed prints.

4. mSLAb operation flowchart

Fig. S5 shows a flowchart for the operation of the mSLAb printer. In addition to the video of exemplary prints (see data repository), including printer operation and sample handling, this flowchart provides a condensed step-by-step overview of the process from printer start-up to starting a print.

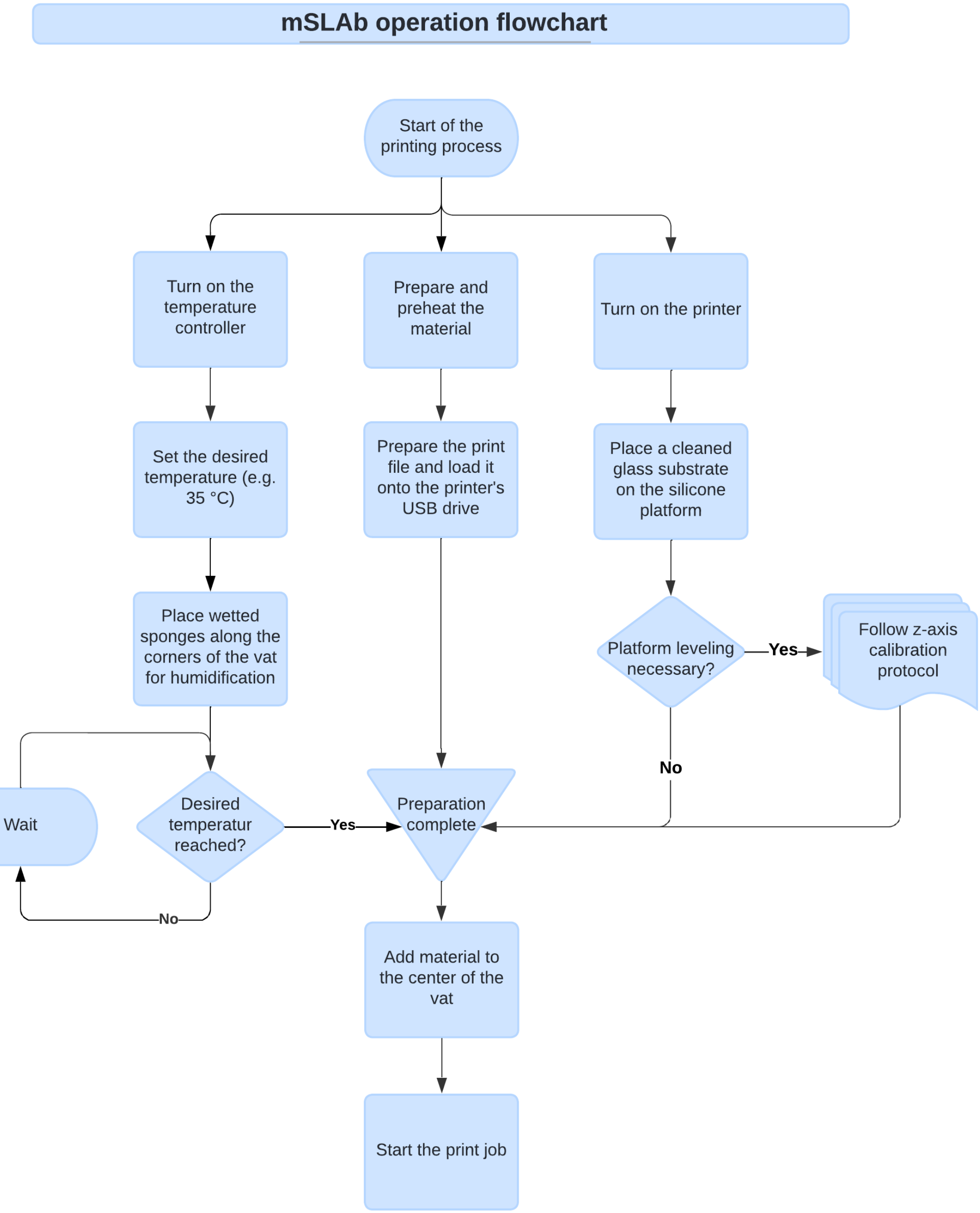

Fig. S5: The mSLAb operation flowchart provides a supplementary step-by-step overview of the process from printer start-up to starting a print. Please refer to the video demonstration in the data repository and the detailed operation instructions in the main manuscript for further information.

[1] C. Claaßen *et al.*, "Quantification of Substitution of Gelatin Methacryloyl: Best Practice and Current Pitfalls," *Biomacromolecules,* vol. 19, no. 1, pp. 42-52, 2018/01/08 2018, doi: 10.1021/acs.biomac.7b01221.
